# Accuracy of IOL Power Calculation Formulas for AcrySof SN60WF versus Tecnis ZCB00 Intraocular Lenses

**DOI:** 10.18502/jovr.v17i3.11571

**Published:** 2022-08-15

**Authors:** Cynthia C Jiang, Noah M Hodson, Daniel A Johnson, Ahmad Kheirkhah

**Affiliations:** ^1^Department of Ophthalmology, University of Texas Health San Antonio, San Antonio, Texas, USA

**Keywords:** Formula Accuracy, Intraocular Lens, SN60WF, ZCB00

## Abstract

**Purpose:**

To compare the accuracy of various intraocular lens power formulas for two monofocal hydrophobic foldable lenses, the AcrySof SN60WF and the Tecnis ZCB00.

**Methods:**

This retrospective study included 409 eyes from 409 patients who underwent uncomplicated cataract surgery (299 eyes with SN60WF and 110 eyes with ZCB00). Biometry was performed for all eyes with an IOLMaster 700. Predicted refraction from five different IOL power formulas (Barrett Universal II, Haigis, Hoffer-Q, Holladay 2, and SRK/T) was compared to postoperative refraction at one to three months for the following axial length strata: short eyes ( < 22.5 mm), medium eyes (22.5–25.5 mm), and long eyes ( > 25.5 mm).

**Results:**

In patients with medium eyes, there were no significant differences in the mean absolute error (MAE) and the percentage of eyes within ± 0.5 D (% ± 0.5 D) between both IOLs. In short eyes, although MAE was similar between both lenses, % ± 0.5 D was significantly higher for Barrett Universal II in ZCB00 than in SN60WF (*P* = 0.01) while Hoffer-Q and Holladay 2 performed equally for both lenses. In long eyes, ZCB00 had a higher MAE than SN60WF for Barrett Universal II, Haigis, and Hoffer-Q. Additionally, in long eyes, the percentage of eyes within % ± 0.5 D was significantly higher for SN60WF than ZCB00 for all formulas (*P* < 0.001).

**Conclusion:**

Although there were no significant differences in the formula accuracy between these two lenses in medium eyes for all formulas and in short eyes for most formulas, the accuracy decreased significantly in long eyes for ZCB00 compared to SN60WF. The effect of IOL model on the postoperative outcomes should be further investigated.

##  INTRODUCTION

Cataract surgery is now considered a form of refractive surgery. There is an increasing demand by patients to have optimal uncorrected distance visual acuity postoperatively. In fact, it has been shown that such vision is an important determinant of patient's satisfaction after cataract surgery.^[1]^ To achieve this goal, it is critical to have a postoperative refraction which is as close as possible to the target refraction. This is largely dependent on choosing an intraocular lens (IOL) with the most accurate power.^[2,3]^ To obtain such IOL power, precise biometry as well as an accurate formula for IOL power calculation are required. However, there still remains the question of which formula provides the most consistent and accurate refractive outcome for each individual eye.

Many formulas have been developed to calculate the IOL power.^[4,5,6,7]^Each of these formulas takes into consideration certain variables of the eye, such as corneal power, axial length, anterior chamber depth, or effective lens position, in order to predict the power of the lens.^[8,9]^ The parameters utilized in these equations vary by each formula which is why accuracy of these formulas varies widely.^[10,11,12,13]^ Furthermore, it has been shown that physical and biometric characteristics of the eye can affect the accuracy of different IOL power formulas to different degrees.^[14,15]^ For example, there have been several studies that have investigated the effects of abnormal values of different parameters, such as long and short eyes, on the accuracy of IOL power calculations. ${}^{\left[16--20\right]}$ Although many studies have evaluated the effects of ocular characteristics on the accuracy of IOL power, the possible effects of the type of IOL on this accuracy remains unknown. This is critical as different IOLs have different physical characteristics.^[21,22]^ Multiple previous studies relied on a single type of IOL,^[19,23,24,25]^ or grouped multiple IOLs into a single dataset.^[14,18,26,27]^ In addition, it remains unclear whether some formulas play better for some lenses than for others.

To evaluate the possible effects of the IOL type on the accuracy of IOL power formulas, in this study we compared this accuracy for two different models of IOL. For this, we used two aspheric monofocal hydrophobic acrylic lenses, the AcrySof SN60WF (Alcon Laboratories, Inc., Fort Worth, TX, USA), and the Tecnis ZCB00 (Johnson & Johnson Vision, Santa Ana, CA, USA), both of which are commonly used in cataract surgery.

##  METHODS

This retrospective study included 409 eyes from 409 patients who had cataract surgery with implantation of either AcrySof SN60WF or Tecnis ZCB00. The study was performed at UT Health San Antonio, San Antonio, Texas, USA. The study protocol was approved by the Institutional Review Board, and the study was compliant with tenets of the Health Insurance Portability and Accountability Act (HIPAA) of 1996.

Inclusion criteria consisted of adult patients who had undergone uncomplicated cataract surgery. In patients with bilateral cataract surgery, only data from the eye with the earliest surgery were used. We included only eyes with available data on electronic medical records regarding the preoperative IOL power calculation for all formulas studied and postoperative manifest refraction at one to three months after surgery. We excluded eyes with corneal or anterior segment disease or trauma. We also excluded patients with previous cornea- or lens-based refractive surgery.

For each patient, demographics, ocular biometric values, the power of the IOL used in surgery, and the one-to-three-month postoperative manifest refraction were collected from electronic medical records. All patients had biometry performed with an IOLMaster 700 (Carl Zeiss Meditec AG). The following biometric values were collected: axial length, lens thickness, anterior chamber depth, white-to-white diameter, and keratometry. Furthermore, we gathered the IOL power measured by five different IOL power formulas. The formulas and optimized IOL constants for SN60WF and ZCB00, respectively, assessed in this study were Barrett Universal II (lens factor = 1.88 and 2.09), Haigis (A0 = –0.769 and –1.302; A1 = +0.234 and +0.210; A2 = +0.217 and +0.251), Hoffer-Q (pACD = 5.64 and 5.80), Holladay 2 (ACD constant = 5.601 and 5.786), and SRK/T (A = 119.00 and 119.30).

To evaluate the accuracy of each formula, we calculated the mean numerical error (MNE) and the mean absolute error (MAE). The MNE was defined as the average of actual postoperative refraction minus the predicted refraction by the IOL power formula used for the eye (actual refraction – predicted refraction).^[28]^ The MAE was defined as the average of the absolute values of actual postoperative refraction minus the predicted refraction for each formula. Moreover, we also determined the percentage of eyes within 0.25 D (% ± 0.25 D), 0.5 D (% ± 0.5 D), 1.0 D (% ± 1.0 D), and 2.0 D (% ± 2.0 D) of the predicted refraction for each formula. We further stratified the eyes into subgroups based on the axial length. These subgroup strata were as follows: short eyes ( < 22.5 mm), medium eyes (22.5-25.5 mm), and long eyes ( > 25.5 mm). Then, the aforementioned accuracy parameters were evaluated in each subgroup of axial length. No adjustment was performed for eyes with long axial length.

### Statistical Analysis

Statistical analysis was performed using SPSS (IBM, version 25). Shapiro–Wilk test was first conducted to evaluate the normality of data distribution. Different qualitative and quantitative parameters were compared between SN60WF and ZCB00 groups using Chi-square and Mann–Whitney tests, respectively. Friedman test followed by post hoc Bonferroni test was performed to compare the MNE and the MAE among different formulas for each lens. Percentage of eyes within a specific dioptric range postoperatively was compared between the two lenses using Chi-square and among different formulas for each lens using Cochran's Q test.

##  RESULTS

This study included 409 eyes from 409 patients (253 women and 156 men) with a mean age of 70.6 ± 8.6 years (range, 41–91 years). There were 299 eyes with SN60WF and 110 eyes with ZCB00. There were no significant differences between the two groups regarding demographics or ocular biometric values [Table 1]. However, postoperative refraction was significantly different between the two lenses (–0.17 ± 0.55 D for SN60WF vs –0.01 ± 0.51 D for ZCB00, *P* = 0.02).

Table 2 demonstrates the MAE and the MNE for each formula for both SN60WF and ZCB00 lenses. For the MAE, there were no significant differences between both IOLs for any formula. Although there were no significant differences in the MAE among various formulas for SN60WF, such difference reached statistical significance for ZCB00, with SRK/T having the highest MAE and Barrett Universal II and Holladay 2 showing the lowest MAE (*P* < 0.01). For the MNE, there were no significant differences between both IOLs for all formulas except for Hoffer-Q in which the MNE was significantly lower for SN60WF compared to ZCB00 (*P* = 0.04). For SN60WF, there were significant differences in MNE among various formulas with Barrett Universal II and Holladay 2 having the highest and lowest MNE, respectively (*P* < 0.01). For ZCB00, the difference in the MNE among various formulas was statistically significant and the highest and lowest MNE was seen in SRK/T and Holladay 2 formulas, respectively (*P* < 0.01).

Table 3 shows the percentage of eyes within ± 0.25 D, ± 0.5 D, ± 1.0 D, and ± 2.0 D of the predicted refraction for various formulas for both lenses. There were no statistically significant differences between the SN60WF and ZCB00 lenses for percentage of eyes within ± 0.25 D, ± 0.5 D, ± 1.0 D, or ± 2.0 D of the predicted refraction (all *P*-values > 0.05). For both SN60WF and ZCB00, there were statistically significant differences in the percentage of eyes within ± 0.25 D, ± 0.5 D, and ± 1.0 D, but not ± 2.0 D, among various formulas. For % ± 0.5 D, the highest percentage was seen by Barrett Universal II for SN60WF (*P* < 0.001) and by Barrett Universal II and Holladay 2 for ZCB00 (*P* = 0.02).

### Effects of the Axial Length on the Formula Accuracy

The MAE and percentage of eyes within ± 0.5 D for each formula across different axial lengths for both IOLs have been shown in Tables 4 and 5, respectively.

**Table 1:** Patient demographics and ocular biometric values for eyes with SN60WF and ZCB00 lenses.


	**SN60WF (** ***n*** ** = 299)**	**ZCB00 (** ***n*** ** = 110)**	***P*** **-value**
Age (yr)	70.71 ± 8.60	70.41 ± 8.63	0.57
Sex (F/M)	178/121	75/35	0.14
IOL power used in surgery (D)	20.31 ± 3.21	20.59 ± 3.32	0.46
Axial length (mm)	23.85 ± 1.11	23.90 ± 1.07	0.74
Lens thickness (mm)	4.61 ± 0.41	4.57 ± 0.41	0.38
Anterior chamber depth (ACD)	3.12 ± 0.39	3.17 ± 0.34	0.25
White-to-white diameter (mm)	11.97 ± 0.44	12.00 ± 0.51	0.35
Mean keratometry (D)	43.88 ± 1.58	43.76 ± 1.69	0.48
Postoperative refraction (D)	–0.17 ± 0.55	–0.01 ± 0.51	0.02
	
	

**Table 2:** Mean absolute error (MAE) and mean numerical error (MNE) for each formula for both SN60WF and ZCB00 lenses.


**Formula**	**Mean Absolute Error (MAE)**			**Mean Numerical Error (MNE) **		
	**SN60WF**	**ZCB00**	***P*** **-value**	**SN60WF**	**ZCB00**	***P*** **-value**
Barrett Universal II	0.41 ± 0.37	0.42 ± 0.37	0.94	0.21 ± 0.52	0.28 ± 0.48	0.21
Haigis	0.42 ± 0.38	0.46 ± 0.38	0.24	0.14 ± 0.55	0.20 ± 0.56	0.30
Hoffer-Q	0.44 ± 0.38	0.48 ± 0.39	0.43	0.15 ± 0.56	0.29 ± 0.55	0.04
Holladay 2	0.42 ± 0.36	0.41 ± 0.36	0.69	0.09 ± 0.54	0.18 ± 0.51	0.23
SRK/T	0.49 ± 0.36	0.49 ± 0.66	0.66	0.15 ± 0.54	0.31 ± 0.76	0.07
${}^{*}$ P-value	0.27	0.002	< 0.001	< 0.001	
	
	
${}^{*}$ This *P*-value is for comparison of different formulas in each lens.						

**Table 3:** Percentage of eyes within ± 0.25 D, ± 0.5 D, ± 1.0 D, and ± 2.0 D of the predicted refraction for both SN60WF and ZCB00 lenses.


**Formula**	**SN60WF**				**ZCB00**			
	** ± 0.25 D**	** ± 0.5 D**	** ± 1.0 D**	** ± 2.0 D**	** ± 0.25 D**	** ± 0.5 D**	** ± 1.0 D**	** ± 2.0 D**
Barrett Universal II	40.8%	70.6%	93.0%	99.7%	40.9%	68.2%	91.8%	100.0%
Haigis	41.5%	66.9%	91.6%	99.3%	39.1%	64.6%	89.1%	100.0%
Hoffer-Q	35.8%	65.9%	93.0%	99.7%	33.6%	66.4%	86.4%	99.1%
Holladay 2	40.5%	66.6%	92.6%	99.7%	43.6%	68.2%	92.7%	100.0%
SRK/T	38.5%	67.6%	93.0%	99.7%	37.3%	66.4%	90.9%	98.2%
${}^{*}$ *P*-value	< 0.001	< 0.001	0.01	0.41	< 0.001	0.02	0.001	0.17
	
	
*This *P*-value is for comparison of different formulas in each group.								

**Table 4:** Mean absolute error (MAE) for SN60WF and ZCB00 lenses in short, medium, and long eyes.


**Formula**	**Short eyes (AL: < 22.5 mm)**			**Medium eyes (AL: 22.5–25.5 mm)**			**Long eyes (AL: > 25.5 mm)**		
	**SN60WF**	**ZCB00**	***P*** **-value**	**SN60WF**	**ZCB00**	***P*** **-value**	**SN60WF**	**ZCB00**	***P*** **-value**
Barrett Universal II	0.37 ± 0.26	0.34 ± 0.36	0.78	0.43 ± 0.38	0.40 ± 0.35	0.62	0.35 ± 0.34	0.69 ± 0.50	0.04
Haigis	0.36 ± 0.28	0.53 ± 0.41	0.11	0.44 ± 0.40	0.43 ± 0.35	0.93	0.36 ± 0.37	0.78 ± 0.57	0.03
Hoffer-Q	0.30 ± 0.24	0.33 ± 0.34	0.75	0.45 ± 0.38	0.45 ± 0.36	0.87	0.53 ± 0.40	0.95 ± 0.64	0.04
Holladay 2	0.30 ± 0.23	0.37 ± 0.38	0.67	0.43 ± 0.38	0.38 ± 0.33	0.31	0.45 ± 0.34	0.77 ± 0.58	0.08
SRK/T	0.36 ± 0.28	0.55 ± 0.51	0.36	0.44 ± 0.37	0.48 ± 0.68	0.48	0.41 ± 0.31	0.70 ± 0.52	0.21
*P*-value*	0.48	0.13	0.3	0.005	< 0.001	0.04	
	
	
${}^{*}$ This *P*-value is for comparison of different formulas in each axial length group.									

**Table 5:** Percentage of eyes within ± 0.5 D of the predicted refraction for SN60WF and ZCB00 lenses for short, medium, and long eyes.


**Formula**	**Short eyes (AL: < 22.5 mm)**			**Medium eyes (AL: 22.5–25.5 mm)**			**Long eyes (AL: > 25.5 mm)**		
	**SN60WF**	**ZCB00**	***P*** **-value**	**SN60WF**	**ZCB00**	***P*** **-value**	**SN60WF**	**ZCB00**	***P*** **-value**
Barrett Universal II	70.0%	85.7%	0.001	69.6%	68.8%	0.87	81.8%	42.9%	< 0.001
Haigis	70.0%	57.1%	0.05	65.6%	66.7%	0.88	77.3%	42.9%	< 0.001
Hoffer-Q	83.3%	85.7%	0.55	64.4%	67.7%	0.55	59.1%	28.6%	< 0.001
Holladay 2	76.7%	71.4%	0.33	66.0%	70.8%	0.44	59.1%	28.6%	< 0.001
SRK/T	73.3%	57.1%	0.01	66.4%	68.8%	0.65	72.7%	42.9%	< 0.001
P-value*	0.02	0.16	< 0.001	0.04	0.004	0.41	
	
	
${}^{*}$ This *P*-value is for comparison of different formulas for each lens in each AL group.									

### SN60WF lenses

In short and medium eyes, the MAE was similar among various formulas. In long eyes, however, the MAE was significantly different among various formulas (*P* < 0.001), with Barrett Universal II having the lowest and Hoffer-Q having the highest values. Regarding % ± 0.5 D, there were significant differences among formulas across all axial lengths. The highest percentage of eyes ± 0.5 D from the predicted refraction was seen with Hoffer-Q in short eyes and by Barrett Universal II in medium and long eyes. On the other hand, the lowest % ± 0.5 D value was seen by Barrett Universal II and Haigis in short eyes, by Hoffer-Q in medium eyes, and by Hoffer-Q and Holladay 2 in long eyes.

### ZCB00 lenses

In short eyes, the MAE was similar among various formulas. In medium and long eyes, however, the MAE was significantly different among various formulas (*P* < 0.05). The lowest and highest values of MAE were obtained by Holladay 2 and SRK/T, respectively, in medium eyes, and by Barrett Universal II and Hoffer-Q, respectively, in long eyes. Although there were no significant differences in the percentage of eyes within ± 0.5 D in short and long eyes, this value was significantly lower in long eyes compared with short and medium eyes for all formulas (all *P*-values < 0.05). In medium eyes, there were significant differences in % ± 0.5 D among various formulas, with Holladay 2 having the highest and Haigis having the lowest values. For all formulas, the % ± 0.5 D was significantly different among short–medium–long eyes (all *P* < 0.05).

### SN60WF versus ZCB00 lenses 

In short and medium eyes, the MAE was similar between SN60WF and ZCB00. However, in long eyes, the MAE was significantly higher in ZCB00 compared with SN60WF for Barrett Universal II (*P* = 0.04), Haigis (*P* = 0.03), and Hoffer-Q (*P* = 0.04). In medium eyes, the % ± 0.5 D for ZCB00 and SN60WF was similar. However, in short eyes, although there were no statistically significant differences in % ± 0.5 D between both lenses for Haigis, Hoffer-Q, and Holliday 2, ZCB00 had a higher % ± 0.5 D for Barrett Universal II (*P* = 0.01) while SN60WF had a higher % ± 0.5 D for SRK/T (*P* = 0.01). In long eyes, SN60WF had a higher % ± 0.5 D than ZCB00 for all formulas (*P* < 0.001 for all formulas).

##  DISCUSSION

In this study, we evaluated the accuracy of five IOL power formulas for two aspheric monofocal hydrophobic acrylic foldable IOLs, the AcrySof SN60WF, and Tecnis ZCB00. We found that the accuracy of the IOL power formulas for these two lenses varied based on the eye's axial length. Although there were no significant differences in the accuracy between these two lenses in medium eyes, the accuracy decreased in short and especially long eyes where postoperative refractive predictability was different for the SN60WF and the ZCB00. Furthermore, relative accuracy of each formula compared to others was also dependent on the axial length and IOL type.

Although the accuracy of IOL power calculation formulas and the role of ocular parameters in such accuracy have been well studied, there are limited data on the effects of IOL model on the accuracy. Melles et al^[28]^ compared two lenses from the same manufacturer, AcrySof SN60WF to SA60AT (Alcon), and found similar rankings of seven IOL formulas (Barrett Universal II, Haigis, Holladay 1, Holladay 2, Hoffer-Q, Olsen, and SRK/T, and Hoffer-Q) between these two lenses. In our study, however, we compared the IOL power formula accuracy between two aspheric monofocal acrylic hydrophobic lenses from two different manufacturers as it has been shown that they have different physical characteristics and visual performance.^[29,34,35,36]^

For evaluation of the accuracy of IOL power formulas in this study we used the MAE, the MNE, and the percentage of eye within ± 0.5 D of the predicted refraction, as reported before.^[37]^ Gale et al^[30]^ established the benchmark standards for refractive outcomes as percentage of eyes within ± 1.0 D of the predicted refraction > 85% and percentage of eyes within ± 0.5 D of the predicted refraction > 55%. Our data indicates this benchmark was met for all formulas for all ranges of axial length in SN60WF lenses. For ZCB00 lenses, however, this benchmark was met for all formulas only for short and medium eyes, but not for any formula in long eyes, showing the effects of IOL model on the accuracy of IOL power formulas.

Although the difference in the accuracy of IOL power formulas between the two lenses was less evident when all axial lengths were taken together, the difference was more prominent when evaluating eyes with different axial lengths separately. In medium eyes with an axial length of 22.5–25.5 mm, the MAE and the percentage of eyes within ± 0.5 D were similar between SN60WF and ZCB00 for any formula. In short eyes ( < 22.5 mm), however, although there was no significant difference between the lenses in the MAE, the percentage of eyes within ± 0.5 D was different between the two lenses for some formulas such as Barrett Universal II in which this formula performed better for ZCB00 than for the SN60WF. The difference between the two IOLs was most remarkable in long eyes with an axial length > 25.5 mm. In these eyes, the MAE was significantly higher for ZCB00 compared with SN60WF for most of the formulas evaluated showing a potential error in IOL power calculation in this IOL model. In addition, the percentage of eyes within ± 0.5 D was significantly higher for SN60WF compared with ZCB00 for all formulas. Although the reasons for such performance of IOL power formulas for ZCB00 lenses in long eyes remain to be determined, adjustment of IOL power formulas may be considered for such situations.

It is well known that IOL power formulas have different accuracies for eyes with different axial lengths. ${}^{\left[14,27\right]}$ For all axial lengths combined, some studies have indicated that Barrett Universal II has a significantly lower MAE and highest percentage within ± 0.25 D, ± 0.5 D, or ± 1.0 D of the target refraction than Haigis, Hoffer-Q, Holladay 1, Holladay 2, SRK/T, and T2 for SN60WF,^[24]^ and a lower MAE and highest percentage within ± 0.25 D, ± 0.75 D, or ± 1.0 D of the target refraction than Holladay 2, Hoffer-Q, and SRK/T for ZCB00.^[32]^ However, our findings showed that formula performance is also dependent on the IOL type. In fact, such accuracy can affect our choice of IOL implant for each individual eye.

In medium eyes in our study, for SN60WF there was no significant difference in the MAE between formulas, but Barrett Universal II had the highest percentage (69.6%) of eyes within ± 0.5 D of the predicted refraction. For ZCB00 lenses, in contrast, Holladay 2 formula was associated with the lowest MAE and the highest percentage (70.8%) of eyes within ± 0.5 D. Aristodemou et al^[31]^ found similar accuracy for eyes implanted with either LI61AO Sofport or Akreos Fit (Bausch & Lomb, Rochester, NY) with axial length between 22.50 and < 25.00 mm when comparing the MAE between Hoffer-Q, Holladay 1, and SRK/T. Narváez et al^[33]^ similarly did not find a difference between formulas (Hoffer-Q, Holladay 1, Holladay 2, and SRK/T) in average (22.0 to < 24.5 mm) and medium-long (24.5 to < 26.0 mm) eyes implanted with either CC4204BF Collamer plate-haptic IOL or AA4203VF silicone plate-haptic IOL (STAAR Surgical, Monrovia, CA). Notably, neither of those studies included Barrett Universal II or compared SN60WF and ZCB00 lenses. Tang et al^[40]^ found no significant difference in accuracy for short ( < 22.0 mm), medium (22.0–25.0 mm), or long ( > 25.0 mm) eyes implanted with SN60WF between Holladay 2, Barrett Universal II, and Hill-RBF formulas. Kane et al^[24]^ found statistically significant differences in the MAE among seven different formulas for their established ranges of medium (22.0 to < 24.5 mm) and medium-long (24.5 to < 26.0 mm) eyes implanted with SN60WF. For both groups, Barrett Universal II had the lowest MAE, and SRK/T performed better than both Haigis and Hoffer-Q formulas for medium eyes but not medium-long eyes.^[24]^ Barrett Universal II additionally had a significantly higher percentage within ± 0.25 D, ± 0.5 D, ± 1.0 D, or ± 2.0 D for medium eyes.^[24]^ As previous studies have used only one IOL model or considered all IOL models together, no comparison can be made with our findings.

For short eyes with axial length of < 22.5 mm, we did not find any significant difference in the MAE and the percentage within ± 0.5 D between the five formulas for ZCB00 lenses. For SN60WF lenses, although there was no significant difference in the MAE for different formulas, the percentage within ± 0.5 D was significantly higher in Hoffer-Q (83.3%). Previously, it has been shown that neither Hoffer-Q, Holladay 2, nor SRK/T consistently outperformed one another in eyes implanted with Acrysof SN60WF or SA60AT (Alcon Laboratories, Inc., Fort Worth, TX) or LI61AO Sofport or Akreos Fit (Bausch & Lomb, Rochester, NY) with an axial length < 22.0 mm.^[28,31]^ In comparing the accuracy of Barrett Universal II, Haigis, Hoffer-Q, Holladay 1, Holladay 2, SRK/T, and T2 formulas, Kane et al^[24]^ did not find any significant difference in formulas with an axial length < 22.0 mm in eyes implanted with SN60WF. A 2017 meta-analysis investigating the accuracy of six IOL power formulas (Haigis, Hoffer-Q, Holladay 1, Holladay 2, SRK/T and SRK II) in short eyes (axial length < 22.0 mm) demonstrated that although Holladay 2 offered the smallest MAE (not statistically significant), Haigis may be superior to Hoffer-Q (*P* = 0.003), SRK/T (*P* = 0.009), and SRK/T II (*P* = 0.01) for short eyes.^[38]^ The IOLs used in this meta-analysis included SA60AT, MA60 (Alcon Laboratories, Inc., Fort Worth, TX), LI61A0 Sofport/Akreos Fit, AMO Sensar AR40e and Tecnis ZA9003 (Johnson & Johnson Vision, Santa Ana, CA), ACR6D (Laboratoires Cornéal, Paris, France), Hoya PY-60AD (Hoya Corporation, Japan), and SN60WF.^[38]^

For long eyes with an axial length of > 25.5 mm, we noted that Barrett Universal II formula had the lowest MAE compared with other four formulas for both ZCB00 and SN60WF lenses. Barrett formula also had the highest percentage (81.8%) of eyes within ± 0.5 D of the predicted refraction for SN60WF lenses. Although there were no significant differences between the percentage of eyes within ± 0.5 D among the five formulas for ZCB00 lenses in long eye, the values were significantly less compared with short and medium eyes. A 2018 systematic review and meta-analysis investigating the accuracy of six IOL power formulas (Barrett Universal II, Haigis, Hoffer-Q, Holladay 1, Holladay 2, and SRK/T) for long eyes with an axial length of > 24.5 mm found that Barrett Universal II not only had a significantly lower MAE than Holladay 1, Holladay 2, Hoffer-Q, and SRK/T (all *P* < 0.001), but had significantly higher percentage of eyes within ± 0.5 D of the predicted refraction than all formula.^[39]^ IOLs used in this study included LI61A0 Sofport/Akreos Fit, SN60WF, SA60AT, PY-60AD, AR40e, and SN60AT and MA60AC/BM/MA.^[39]^ Kane et al^[24]^ also found Barrett Universal II to be the most accurate for eyes with an axial length ≥ 26.00 mm for SN60WF. Kane et al^[24]^ found long eyes implanted with SN60WF to have formulas with much lower percentage of eyes within target refraction compared to medium or medium-long axial lengths; however, from our study this was only true for ZCB00. In our study, postoperative refractive predictability was significantly better for the SN60WF than the ZCB00 in long eyes with a higher percentage within ± 0.5 D for all formulas: for example, 81.8% versus 42.9%, respectively, for Barrett Universal II formula.

Although SN60WF and ZCB00 lenses are commonly used during cataract surgery, a potential limitation to our study is that we did not evaluate other models of IOLs, including those with other materials such as hydrophilic acrylic or silicone IOLs. Furthermore, eyes with previous surgeries, including keratorefractive surgery, were excluded from our study. The differential effects of these surgeries on the accuracy of IOL power formulas for various lenses remain to be determined. Newer IOL power formulas, such as Kane, Olson, and Hill-Radial Basis Function (RBF), may also have different accuracies compared to the formulas evaluated in our study for different IOL models. Moreover, although many different methods of adjustments have been suggested for the long eyes, such as axial length adjustment,^[41]^ we did not apply any of these adjustments in this study as it is unknown whether such adjustments are dependent on the IOL model.

In summary, our study showed that the accuracy of IOL power formula can be different for AcrySof SN60WF or Tecnis ZCB00. As such, difference may be true for other IOL models, studies on the IOL formula accuracy should report the results for each IOL model separately and should avoid combining different IOL models into one group. Different adjustments may be required for various IOL types in different axial lengths. Although significant differences in lens performance were not seen in medium eyes, cataract surgery in short or particularly long eyes should prompt deliberation in choice of IOL.

##  Financial Support and Sponsorship

None.

##  Conflicts of Interest

None declared.**
